# Comparison of the Video Game Functional Assessment-Revised (VGFA-R) and Internet Gaming Disorder Test (IGD-20)

**DOI:** 10.3389/fpsyg.2019.00310

**Published:** 2019-02-19

**Authors:** Matthew Evan Sprong, Mark D. Griffiths, Daniel Perry Lloyd, Erina Paul, Frank D. Buono

**Affiliations:** ^1^School of Interdisciplinary Health Professions, Northern Illinois University, DeKalb, IL, United States; ^2^Psychology, Nottingham Trent University, Nottingham, United Kingdom; ^3^Department of Psychiatry, School of Medicine, Yale University, New Haven, CT, United States; ^4^Center for Molecular Medicine and Genetics, Wayne State University School of Medicine, Detroit, MI, United States

**Keywords:** internet gaming disorder, video game addiction, DSM-5, video game functional assessment, internet gaming addiction, internet gaming addiction (IGA)

## Abstract

Initially labeled as internet addiction in the mid-1990s (e.g., Griffiths, 1996; Young, 1996), researchers have since focused on how specific online activities result in negative consequences for those who overuse and have problems with online applications such as online gambling and online sex (Griffiths, 2000; Potenza, 2017). More recently, this has been applied to online problematic video game play, often used synonymously with terms such as online video game addiction, online gaming addiction, and Internet gaming disorder (IGD). With the publication of the fifth edition of the Diagnostic and Statistical Manual of Mental Disorders (DSM-5; American Psychiatric Association [APA], 2013), IGD was identified by the APA as warranting further study. The current proposed diagnostic criterion in the DSM-5 requires the presence of five of nine symptoms over a 12-month period. These include: (a) preoccupation or obsession with Internet games, (b) withdrawal symptoms when not playing Internet games, (c) an increasing need over time to spend more and more time playing video games, (d) failed attempts to stop or curb Internet gaming, (e) loss of interest in other activities such as hobbies, (f) continued overuse of Internet games even with knowledge of the impact of overuse on their life, (g) lying about extent of Internet game usage, (h) uses Internet games to relieve anxiety or guilt, and (i) has lost or put at risk an opportunity or relationship because of Internet games (American Psychiatric Association [APA], 2013). However, it is unclear if the disorder represents addiction to the internet or if IGD evaluates specific behaviors occurring within the context of the video gaming (Starcevic and Billieux, 2017; Young and Brand, 2017).

## Introduction

To evaluate the diagnostic utility of the prosed DSM-5 criteria Pontes and Griffiths (2014) developed the 20-item Internet Gaming Disorder Test (IGD-20), a brief questionnaire based on an addiction components model (Griffiths, 2005). Griffiths stated that addiction must be understood in terms of core characteristics that occur in both problematic use of substances and behaviors (salience, mood modification, tolerance, withdrawal, conflict, and relapse). Pontes et al. compared these sub-factors in a large sample of gamers to the DSM-5 criteria of IGD and found the entire assessment to have good reliability and validity. Moreover, the IGD assessment was shown to correspond within criterion established for the DSM-5 definition of IGD [i.e., Salience – Criterion (a), Mood Modification – Criterion (h), Tolerance – Criterion (c), Withdrawal – Criterion (b), Conflict – Criteria (e, f, g, i), Relapse – Criterion (d)]. However, the utility of the assessment is limited beyond providing generalized, non-specific treatment recommendations or alerting the individual that their gaming behavior puts them in the danger of developing an addiction. Furthermore, the IGD-20 does not inform the individual at what point gaming becomes problematic and lacks the ability to assist researchers in addressing and reducing the motivation to engage in problematic play.

The field of applied behavior analysis has evaluated the motivation underlying maladaptive behaviors such as pathological gambling, sexual addiction, or problematic video gaming (Cooper et al., 2007; Vollmer et al., 2015). This research asserts motivation is typically maintained by providing the individuals with at least one of the following functions: (i) social attention, (ii) tangible/intangible rewards, (iii) escape/avoidance of demands or pain, and (iv) sensory stimulation. Through functional analysis of the antecedents and consequences of a given behavior, it becomes possible to assess the motivation and isolate the main function of a maladaptive, isolating, or undesirable behavior. These are ‘paper-and-pencil’ tasks where individuals rank targeted behaviors via clear, simplistic structured sentences. The Video Game Functional Assessment-Revised (VGFA-R; Buono et al., 2016) was designed and is the only assessment to evaluate the reinforcing behavioral motivation of video game players by assessing the function of their video game play. More recently, Buono et al. (2017) found individuals reporting “high” levels of play (e.g., 24 h of gaming per week and above) were largely motivated by the escape/avoidance or social attention functions. While effective at evaluating an individual’s motivation for gameplay, further work on the VGFA-R is required to determine if high levels of play meet the criteria of IGD as outlined in the DSM-5.

The diagnostic criteria of IGD encompass those used in potentially addictive gameplay, as well as allied behaviors such as smartphone addiction and problematic internet use (Lopez-Fernandez et al., 2018). Additionally, the criteria share characteristics with other behavioral addictions such as pathological gambling and problematic social media use (Wood et al., 2007; Oggins and Sammis, 2010; Pontes and Griffiths, 2014; Kuss and Griffiths, 2017; Potenza, 2017). Although several treatment modalities based upon the principle of cognitive behavioral therapy are showing initial promise (Torres-Rodriguez et al., 2017a,b; Young and Brand, 2017), there remains a need for rigorous, empirically validated treatments for IGD. It is thus crucial to provide accurate diagnosis and effective, empirically validated treatment of individuals struggling with video game addiction. Therefore, the focus of the present study is to compare the DSM-5 validated assessment criteria of the IGD-20 with the primary reinforcing behavioral functions evaluated by the VGFA-R. More specifically, the study compares the component factors outlined in the IGD-20 (salience, mood modification, tolerance, withdrawal, conflict, and relapse) and the subscales of the VGFA-R (social attention, tangible/intangible rewards, escape/avoidance of demands, and sensory stimulation) by conducting a confirmatory factor analysis (CFA) of video game players at a midwestern university in the United States. By embedding the VGFA-R more firmly with the current proposed DSM-5 criteria for IGD, the present study provides an examination of the potential overlap between behavioral motivation and the formal diagnosis of IGD. Furthermore, we were interested in observing if a direct relationship exists between minutes played in a gaming session and each scale.

## Materials and Methods

### Participants

A total of 320 participants showed initial interest in completing the survey. Of the total number of participants, 304 completed the entire study and had a mean age of 29.82 years (*SD* = 9.82). A total of 178 participants indicated they were female (58.55%) with 126 reporting as male (41.45%). Most of the study participants reported being White Non-Hispanic (*n* = 190, 62.50%). A total of 37 participants reported being Black or African American (12.17%), 23 participants reported being Asian (7.57%), and 30 participants reported being Hispanic or Latino (9.87%). Participants played an average of 13.78 h per week (*SD* = 11.79), and an average of 175.75 min (2.93 h) each time the participant engaged in a gaming session. The average age of when study participants first started gaming was 10.94 years of age (*SD* = 7.54). See Table 1 for other demographic information.

**Table 1:** Demographic information of video gaming participants (*N* = 304).

	*N*	% of Population
**Hours played/week**		
0 to 5	88	28.95
6 to 11	83	27.30
12 to 17	57	18.75
18 to 23	32	10.53
24+	44	14.47
**Relationship status**		
Married/domestic partnership	103	33.88
In a relationship	24	7.89
Single	153	50.33
Divorced/separated	8	2.63
**Type of games played**		
Role-playing	191	34.12
First-person shooter	194	34.19
Real-time strategy	131	22.47
Turn-base	99	24.37
Simulation	187	24.26
Sports	117	15.23
Facebook	154	15.26


For both the Qualtrics’ community engagement tool and recruitment at the United States midwestern school, the identical inclusion and exclusion criteria were utilized. In which, inclusion for the study was active video game players who self-reported playing video games for at least an hour per week, and individuals who were 18 years or older at the time of the study. Exclusion criteria were individuals who did not have access to internet-based computer, tablet, or phone to complete the survey.

### Materials

The VGFA-R is a 24-item Likert-style scale was designed to assess four functions (i.e., attention, escape, tangible, sensory stimulation) that maintain video game play (Buono et al., 2016). Participants were presented with a question (e.g., I choose to play video games when I am nervous or anxious) and were asked to select one of seven responses (1 = Never, 2 = Almost Never, 3 = Seldom, 4 = Half of the time, 5 = Usually, 6 = Almost Always, 7 = Always). Each behavioral function has six questions associated with it, and the scores for each question are combined and may range between 7 and 42, with total scores ranging between 7 and 168. Higher scores indicate the behavioral function is a strong indicator of the motivation for continued video game play. The VGFA-R had strong overall internal consistency (α = 0.927) and across the four functions: attention (α = 0.911), escape (α = 0.796), tangible (α = 0.835), sensory (α = 0.795) (Buono et al., 2016).

The IGD Test is a 20-item Likert-style scale was developed to assess six components of addictive behavior (e.g., salience, mood modification, tolerance, withdrawal symptoms, conflict, and relapse) associated with the DSM-5 criteria for IGD diagnosis (Pontes and Griffiths, 2014). Each component comprises of three to five questions per domain: salience (3), mood modification (3), tolerance (3), withdrawal symptoms (3), conflict (5), and relapse (3). Participants were presented with a question (e.g., I often lose sleep because of long gaming sessions) and were asked to choose one of five responses (1 = strongly agree, 2 = agree, 3 = neither agree nor disagree, 4 = disagree, 5 = strongly disagree). All items were reversed scored with the exception of items 2 and 19, so that a score of 5 was converted to 1, 4 was converted to 2, 2 was converted to 4, and 1 was converted to 5. The IGD had strong overall internal consistency (α = 0.925), and each subscale had good internal consistency, including salience (α = 0.796), mood modification (α = 0.880), tolerance (α = 0.844), withdrawal symptoms (α = 0.921), conflict (α = 0.821), and relapse (α = 0.701).

### Procedure

Approval from the primary author’s institutional review board (IRB) was requested prior to recruiting participants for the study. Once granted (protocol approval code HS17-0060), study materials were developed within the *Qualtrics* online software program. *Qualtrics* is a password-protected online software program that allows a researcher to administer surveys electronically. As a part of other services offered by *Qualtrics*, the community-engaged recruitment feature was utilized for the study. The service was requested by entering information about the study (e.g., characteristics of the study participants we were targeting), the recruitment script and providing the IRB approval form. Participants were provided a recruitment email that disclosed the purpose of the study, the approximate time that it would take to complete the study, participant inclusion information (e.g., above the age of 18 years), information related to discontinuation of the survey, and that they would be reimbursed for their participation.

Additionally, a mass-email was approved by the midwestern university’s IRB to be utilized for recruitment. The identical recruitment script was distributed twice through email by the assistant director of information technology to all actively enrolled college students in the academic year of 2017–2018 in the course of a month. Participants that agreed to participate in the study were instructed to click on the link at the bottom of the recruitment email. The link redirected the study participants to the study materials within *Qualtrics.* The VGFA-R was administered to study participants, followed by demographic information, and concluding with IGD assessment. The materials were administered in this manner because to provide a break between answering questions that have some similarity in phrases. Once participants completed all of the study materials, a debriefing statement was provided and *Qualtrics* paid them directly for their participation.

### Data Analysis

A CFA was performed to assess the relationship between the four functions of the VGFA-R and the six factors of the IGD scales. Previous literature (e.g., Buono et al., 2016, Buono et al., 2017) has already established the functions of the VGFA-R, and Pontes and Griffiths (2014) have cited previous studies that have established the factors of the IGD scale. Holtzman and Vezzu (2011) suggested that once an initial model is established, it is important to perform CFA to confirm that the hypothesized model provides a good fit to the data. If outcome data are collected, such as grades, structural equation modeling (SEM) should also be employed to investigate how well the assessment predicts these measures. It is important to note that the CFA is a part of the SEM. Whether the factor structure of a non-cognitive instrument is determined using psychological theory or empirical research, it is important to perform CFA, which is a special case of what is known as SEM. SEM typically refers to models where causal relationships are investigated between latent variables.

## Results

Given the Exploratory Factor Analysis findings that were reported in several other studies evaluating the VGFA-R (i.e., Buono et al., 2016, 2017) indicating a four-factor solution, a CFA was performed to confirm the hypothesized model was a good fit of data compared to the IGD-20 test. Therefore, we developed four *a priori* factors (i.e., attention, escape, tangible, sensory) for the VGFA-R. Additionally, we developed six *a priori* factors for the IGD-20 (e.g., salience, mood modification, tolerance, withdrawal symptoms, conflict, and relapse) given previous studies that found six factors (e.g., Pontes and Griffiths, 2014).

### Confirmatory Factor Analysis

**FIGURE 1:**
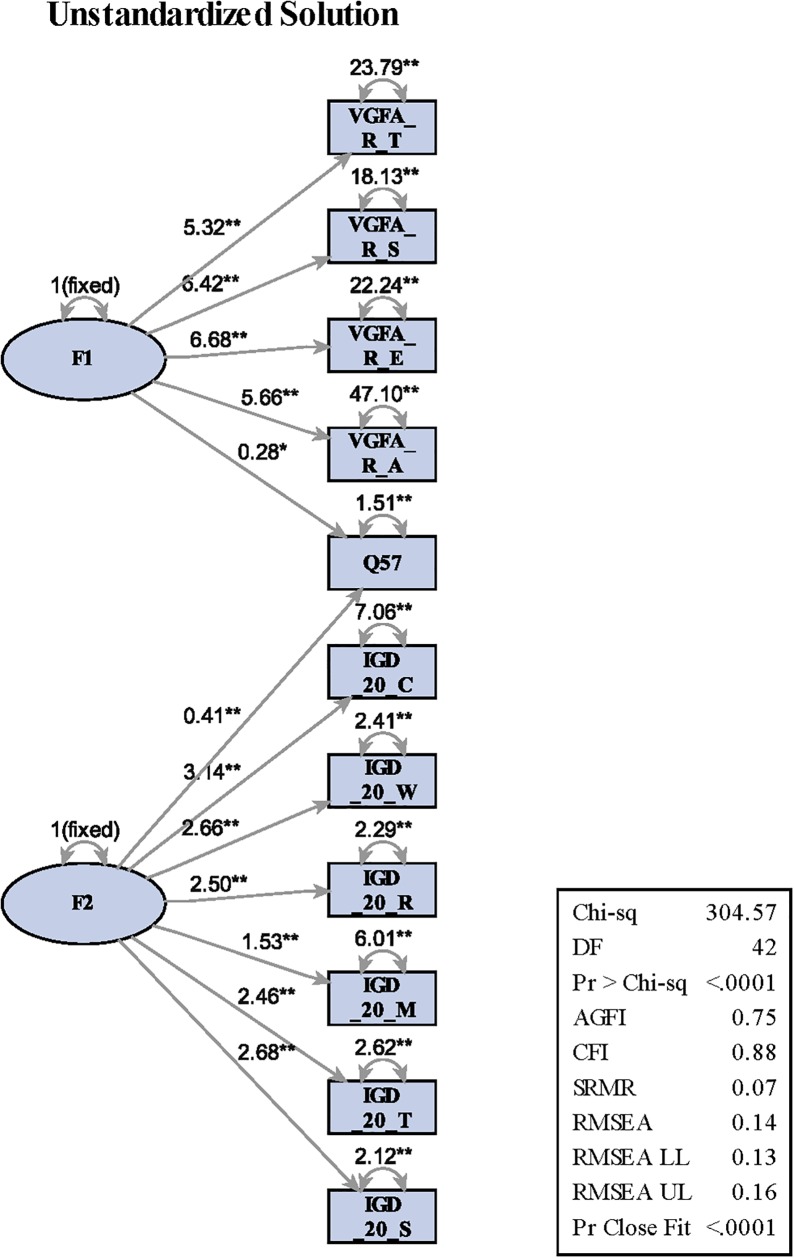
The unstandardized solution for the VGFA-R and IGD-20.

The CFA for the VGFA-R was estimated with maximum likelihood (ML) estimation. Chi-square statistic goodness-of-fit test, χ^2^(34, *N* = 304) = 271.64, *p* < 0.0001; χ^2^/*df* = 7.99; CFI = 0.88; RMSEA = 0.15 (90% confidence interval [CI]; 0.14, 0.17). All factor loadings were significant (ranging from 5.30 to 6.63), and there was no evidence of cross-loading for any indicator. Since the Chi-square statistic is not close to zero and significant, the data would appear to be a weak fit (Holtzman and Vezzu, 2011). However, Chi-square indicators are highly dependent on sample size, thus suggesting that other fit indices be examined. Therefore, other indices were exampled and reported above (e.g., CFI, RMSEA). The unstandardized solution produced a score of 0.68, which indicates a moderate to good relationship between the two scales (see Figure 1). Examination of the residual correlations, which are the differences between the observed and model-implied correlations, did not reveal any problem related to the indicators of the latent variables (Weston and Gore, 2006). The results suggest that the VGFA-R model approached acceptable levels (CFI = 0.88, where 0.90 is needed for acceptable fit), indicating other fit indices needing to be explored. Correlations among the latent variables and factor loadings of the measurement model are presented in Table 2.

**Table 2:** Correlation coefficients between levels of the VGFA-R and IGD-20 test.

		VGFA-revised			
			
		Attention	Escape	Tangible	Sensory
IGD-20 Test	Salience	0.370	0.589	0.412	0.438
	Mood	0.106	0.606	0.298	0.246
	Tolerance	0.343	0.613	0.493	0.517
	Withdrawal	0.278	0.595	0.368	0.406
	Conflict	0.337	0.550	0.282	0.401
	Relapse	0.320	0.538	0.279	0.397


### Structural Model

In order to fully examine the behavioral functions of the VGFA-R, we used SEM to test the relationships among the VGFA-R constructs and the IGD-20. This was performed using PROC CALIS in SAS. Even though previous studies have provided empirical evidence for the application of the VGFA-R in understanding the function that maintains video game play, there is limited research connecting the behavioral function and factors of the IGD-20 that established items consistent with DSM-5 criteria for IGD diagnosis. The VGFA-R demonstrated acceptable fit to the data, χ^2^(42, *N* = 304) = 304.57, *p* < 0.0001; χ^2^/*df* = 7.25; CFI = 0.88; RMSEA = 0.14 (90% confidence interval [CI]; 0.13, 0.16). The structural relationships among the VGFA-R and IGD-20 are depicted in Figure 1. The corresponding linear equations are presented in Table 3.

**Table 3:** Structural equation model linear equations.

Variable	Linear equation
Time played during gaming session (minutes)	= 0.258 × VGFA-R + 0.4088 × IGD-20 + error
**VGFA-R**	
Attention	= 5.6623 × VGFA-R + error
Escape	= 6.6762 × VGFA-R + error
Tangible	= 5.3162 × VGFA-R + error
Sensory	= 6.4199 × VGFA-R + error
**IGD-20**	
Salience	= 2.6818 × IGD-20 + error
Mood modification	= 1.5341 × IGD-20 + error
Tolerance	= 2.4581 × IGD-20 + error
Withdrawal	= 2.6567 × IGD-20 + error
Conflict	= 3.1425 × IGD-20 + error
Relapse	= 2.5031 × IGD-20 + error


### VGFA-R and Minutes Played Each Gaming Session and Hours Played per Week

Several multiple linear regression analyses were performed to determine if each function of the VGFA-R could be predicted from the minutes played in each gaming session and the estimated number of hours played each week. The null hypothesis tested was the regression coefficient (i.e., slope) as equal to zero. The data were screened for missingness and violation of assumptions prior to analysis. There were no missing data. Results of the regression analysis suggested that all of the functions were significantly predicted by minutes played during each gaming session, including attention [*F*(2,311) = 19.47, *p* < 0.001, *r*^2^ = 0.41], escape [*F*(2,311) = 28.765, *p* < 0.001, *r*^2^ = 0.67], tangible [*F*(2,311) = 21.957, *p* < 0.001, *r*^2^ = 0.54], and sensory [*F*(2,311) = 22.412, *p* < 0.001, *r*^2^ = 0.69]. The individual predictors were examined further for attention and indicated that minutes played each gaming session (*t* = 2.44, *p* = 0.015) and hours played each week (*t* = 4.02, *p* = 0.015) were significant predictors in the model. The individual predictors were examined further for the escape function and indicated that minutes played each gaming session (*t* = 2.68, *p* = 0.008) and hours played each week (*t* = 6.16, *p* < 0.001) were significant predictors in the model. The individual predictors were examined further for the tangible function and indicated that minutes played each gaming session (*t* = 0.132, *p* = 0.03) and hours played each week (*t* = 5.51, *p* < 0.001) were significant predictors in the model. The individual predictors were examined further for the sensory function and indicated that minutes played each gaming session (*t* = 3.58, *p* < 0.001) and hours played each week (*t* = 4.54, *p* < 0.001) were significant predictors in the model.

## Discussion

The present study compared the DSM-5 criteria of the 20-item IGD Test (IGD-20) with the behavioral functions evaluated by the 24-item VGFA-R. More specifically, it compared the component factors of the IGD-20 (salience, mood modification, tolerance, withdrawal, conflict, and relapse) with the VGFA-R subscales (social attention, tangible/intangible rewards, escape/avoidance of demands, sensory stimulation) by conducting a CFA of 304 United States student video game players. Findings indicated a significant relationship between the two instruments. Given that the two instruments were designed in completely different ways for potentially different purposes, and the fact that they are so highly correlated suggests that the VGFA-R could be used by clinicians and practitioners as an adjunct to the IGD-20 and provide extra information relating to the motivations underlying the problematic gaming among their clients.

Looking at the individual correlations in Table 2, it is evident that the motivation most correlated with the six IGD-20 criteria is that of escape (all six correlations above 0.53). This confirms previous research showing that escape is often one of the key motivating factors among those experiencing problematic video gaming, especially when used as a coping strategy to forget about other negative experiences in the gamer’s life (e.g., Sattar and Ramaswamy, 2004; Wan and Chiou, 2006a,b; Wood et al., 2007; Hussain and Griffiths, 2009; Griffiths, 2010). The escape motivation was also a significant predictor of the amount of time spent gaming both within-session and across the whole week (i.e., the more the motivation was to escape, the greater amount of time spent gaming within-session and weekly). Although the exact reasoning as to why the duration of video gaming increased for participants scoring high in the escape function category, previous studies (e.g., Kim et al., 2017) have found that individuals that could be classified as having internet game addiction were attempting to escape from negative emotions such as major depressive disorder, dysthymia, and depressive disorders. Previous research has identified that video gaming behavior is due to one of the four behavioral functions as measured by the VGFA-R. For example, Fuster et al. (2013) found that socialization (equivalent to attention function from the VGFA-R), achievement (equivalent to the tangible function from the VGFA-R), and escapism and/or stress relief (equivalent to the escape function from the VGFA-R) were all components related to why people are motivated to play video games. Other studies, such as Hilgard et al. (2013) explored the risk factors associated with pathological game use (defined as excessively frequent or prolonged use) and found three primary factors related to pathological game usage, including (1) the use of games to escape daily life, (2) the use of games as a social outlet, and (3) positive attitudes toward the steady accumulation of in-game rewards (perhaps building a desired character to represent what the gamer wishes their real-life was).

The present study is not without its limitations. The data were self-report and the participants were recruited via convenience sampling that is unlikely to be representative of the United States population or the gamer population. The use of self-report data is known to have a number of well-known biases (most notably biases concerning social desirability and memory recall). However, these are present in all studies using self-report data and readers are advised to take this into account when interpreting the data. The sample size was modest (although acceptable for the kinds of analysis carried out). Future studies should replicate the present study with larger and more representative samples (especially those who are representative of the gaming community rather than a particular country, although cultural differences are likely).

The present study demonstrates that high scores on VGFA-R and the IGD-20 are significantly correlated with each other and each scale provides evidence of construct validity for the other that they are assessing what they are supposed to be assessing. Motivations play an important role in the development of problematic gaming and the present study appears to show that escape is the most important motivational factor in repeat playing. We found that all four motivations in the VGFA-R (i.e., escape, attention, sensory, tangible) were strong predictors of the duration of time a gamer will play in a single gaming session (measured by minutes played each gaming session). Given the findings from previous research and findings from the current study, the VGFA-R may be a useful tool in the development of clinical interventions and further research that investigates methods of reducing undesired play when video gaming becomes problematic.

## Ethics Statement

Ethics Committee: Jeanette Gommel (Research Compliance Coordinator), Office of Research Compliance, Integrity and Safety, Division of Research and Innovation Partnerships. The consent procedure was discussed within the manuscript but participants were recruited in two ways (students at a Midwestern University – a mass email system sent out our recruitment email discussing the study methodologies and their rights to participate, and Qualtrics community engaged recruitment program). Participants were allowed to click on the link if they wished to participate or close their browsers if they did not want to participate. After receiving the recruitment email, they were taken to a consent page where they were again told their rights to participate and discontinue at any time without penalization. We did not know who participated so all data was anonymous. No vulnerable populations participated (we do not know if our participants had disabilities or not because we did not ask).

## Author Contributions

MS and FB designed the study and wrote the methods section. MG assisted with introduction and wrote discussion section. EP and MS analyzed the data. MS wrote up the results section. DL wrote the introduction and conducted literature review.

## Conflict of Interest Statement

The authors declare that the research was conducted in the absence of any commercial or financial relationships that could be construed as a potential conflict of interest.
